# Intra-articular delivery of AAV vectors encoding PD-L1 attenuates joint inflammation and tissue damage in a mouse model of rheumatoid arthritis

**DOI:** 10.3389/fimmu.2023.1116084

**Published:** 2023-03-03

**Authors:** Wenjun Li, Junjiang Sun, Susi Liu Feng, Feng Wang, Michael Z. Miao, Eveline Y. Wu, Shannon Wallet, Richard Loeser, Chengwen Li

**Affiliations:** ^1^ Gene Therapy Center, University of North Carolina at Chapel Hill, Chapel Hill, NC, United States; ^2^ Division of Oral and Craniofacial Biomedicine, University of North Carolina Adams School of Dentistry, Chapel Hill, NC, United States; ^3^ Division of Chemical Biology and Medicinal Chemistry, School of Pharmacy, University of North Carolina at Chapel Hill, Chapel Hill, NC, United States; ^4^ Thurston Arthritis Research Center, The University of North Carolina at Chapel Hill, Chapel Hill, NC, United States; ^5^ Department of Pediatrics, University of North Carolina at Chapel Hill, Chapel Hill, NC, United States; ^6^ Department of oral biology, University of Florida, Gainesville, FL, United States; ^7^ Division of Rheumatology, Allergy, and Immunology, University of North Carolina, Chapel Hill, NC, United States; ^8^ Carolina Institute for Developmental Disabilities, University of North Carolina at Chapel Hill, Chapel Hill, NC, United States

**Keywords:** PD-L1, rheu matoid arthritis, AAV (Adeno-associated virus), Inflammation, Gene Therapy

## Abstract

**Objective:**

Rheumatoid arthritis (RA) is the most common form of autoimmune inflammatory arthritis. Intra-articular gene delivery to block proinflammatory cytokines has been studied in pre-clinical models and human clinical trials. It has been demonstrated that the level of programmed death-ligand 1 (PD-L1) is associated with rheumatoid arthritis (RA). This study examined the therapeutic role of PD-L1 by intra-articular delivery via adeno-associated virus (AAV) vectors in the mouse collagen-induced arthritis (CIA) model.

**Methods:**

Mice were intra-articularly injected with AAV5 vectors encoding human PD-L1 on day 0 and immunized with bovine type II collagen to induce CIA simultaneously. On day 49 post AAV administration, joints were collected for histo-pathological and cytokine analysis. Additionally, the systemic impacts of intra-articular injection of AAV5/PD-L1 vectors were also studied. To study the therapeutic effect of PD-L1, AAV5/PD-L1 vectors were administered into the joints of RA mice on day 21.

**Results:**

After administration of AAV5/PD-L1 vectors, strong PD-L1 expression was detected in AAV transduced joints. Joints treated with PD-L1 at the time of arthritis induction exhibited significantly less swelling and improved histopathological scores when compared to untreated joints. Additionally, the infiltration of T cells and macrophages was decreased in joints of CIA mice that received AAV5/PD-L1 vectors (P<0.05). The levels of pro-inflammatory cytokines, including IL-1, IL-6, IL-17 and TNFα, were lower in AAV5/PD-L1 treated than untreated joints (P<0.05). Furthermore, the administration of AAV5/PD-L1 vectors into the joints of CIA mice did not impact serum cytokine levels and the antibody titers to type II collagen. Biodistribution of AAV vectors after intra-articular injection showed undetectable AAV genomes in other tissues except for a low level in the liver. Similar to the results of AAV5/PD-L1 vector administration on day 0, decreased joint swelling and lower histopathological damage were observed in joints treated with AAV5/PD-L1 vectors on day 21.

**Conclusion:**

The results from this study demonstrate that local AAV mediated PD-L1 gene delivery into the joints is able to prevent the development and block the progression of arthritis in CIA mice without impacting systemic immune responses. This study provides a novel strategy to effectively treat inflammatory joint diseases using local AAV gene therapy by interference with immune checkpoint pathways.

## Introduction

Rheumatoid arthritis (RA) is a chronic inflammatory disease characterized by skewed and dysregulated immune responses that affect multiple organs, particularly the joints. As one of the most common autoimmune disorders, the prevalence rate of RA is approximately 1% worldwide (1), with higher risk of development in middle-aged women (2). In addition to articular joints, RA can impact other tissues, such as blood vessels, heart, or lungs (3). Due to progressive joint inflammation and damage, as well as extra-articular involvement, patients often suffer from pain, impaired mobility, and decreased life expectancy (4).

Currently, there is no perfect cure for RA and patients are treated with one or more disease-modifying antirheumatic drugs (DMARDs) and/or biologics such as cytokine inhibitors to inhibit immune and inflammatory responses. However, less than 30% of patients have had robust responses to these drugs (2), and long-term remission is not achieved for many patients (5). Additionally, these therapeutics can be expensive and have a number of side-effects related to their systemic delivery (6), in particular an increased risk of certain infections (7).

Systemic treatments have been widely applied for management of RA since it is a systemic disease. However, these treatment regimens are not always ideal. First, it is difficult to maintain a satisfactory drug concentration in specific sites where the disease is particularly active. This is supported by the fact that at least 10% of patients with RA still end up with severe disabilities despite undergoing systemic treatments (3). Second, systemic treatments require a much higher dose than local treatments, which results in increased adverse effects. Third, in some patients only one or a few joints may be active and therefore having an option of an effective intra-articular therapy would reduce the need for increasing the dose or changing of systemic therapies.

Even though RA as a systemic disease, demands systemic treatment for full recovery, intraarticular gene therapy, particularly with adeno-associated virus (AAV) vectors, has been explored to address the most severe problem in local sites. Due to the rapid clearance in the synovial space (8), traditional drug therapy has encountered problems with achieving a sustained and therapeutic concentration in affected joints with intra-articular injection and repeated administration of drugs is usually required (9); Gene delivery to the synovium could induce sustained therapeutic gene expression locally within joints depending on the AAV serotypes used (10). It has demonstrated that one-time administration of AAV vectors is able to achieve transgene expression in humans for as long as 10 years (11). There have been several clinical trials using AAV vectors for intra-articular delivery (8), mostly targeting a single cytokine, such as IL-1Ra, IL-6, and TNFα (12, 13), but the benefit has been limited suggesting that other targets are needed.

It has been well known that immune cells infiltrate the joints in inflammatory arthritis, especially T cells and macrophages (14, 15). These immune cells are able to secrete a variety of cytokine/chemokines in the joint (2). In particular, the CD4+ T cell subsets, T helper type 1 cells (Th1), T helper type 17 cells (Th17) and regulatory T cells (Treg), are critical for RA initiation and development (16). Therefore, targeting immune cells may represent a more ideal strategy to improve arthritis. It was reported that therapies to block T cell co-stimulation by targeting CD80/CD86-CD28 interactions were very effective in remediating both early and advanced disease (15, 17, 18). Jun et al. applied anti-human DR5 antibody TRA-8 to deplete macrophages and decreased severity of arthritis (19). These results indicate that the suppression of T cells and/or macrophages is critical for RA treatment.

PD1 is a well-known checkpoint protein for the maintenance of immune cell homeostasis (20). PD1 is expressed on immune cells including T cells and macrophages (21–23). Its ligand PD-L1 is expressed ubiquitously on all cell types and is able to interact with PD1 on immune cells to influence the function of immune responses (15). The binding of PD-L1 to PD1 on immune cells initiates the recruitment of Src Homology 2-Domain-Containing Tyrosine Phosphatase 1 and 2 (SHP-1/SHP-2) (24), that causes the dephosphorylation of signaling kinases such as CD3ζ, PKCθ and ZAP70 (24), and leads to the inactivation of the transcription factors such as STAT family and a global inhibition of immune cells (25). Reports have shown that PD-L1 + cells were significantly altered in synovium tissue of untreated RA patients (26, 27), thus, increasing PD-L1 expression on immune cells from RA patients can be a potential strategy for RA treatment.

In this study, we explored the role of the PD1/PD-L1 pathway in the pathogenesis of arthritis in an RA mouse model. Our results showed that intra-articular injection of AAV vectors encoding PD-L1 was able to prevent the development and block the progression of arthritis in mice with collagen induced arthritis (CIA). The expression of PD-L1 in the joints alleviated knee joint swelling and inflammation by decreasing T cell/macrophage infiltration and proinflammatory cytokine production while maintaining a safe profile without significant systemic side effects.

## Material and methods

### Cells and AAV vector production

HEK-293 cells were cultured in Dulbecco’s Modified Eagle Medium with 10% fetal calf serum, 100 U ml−1 penicillin G and 100 μg ml−1 streptomycin in the 37°C incubator. Cells were routinely split 1:5 three times a week, when ~90% confluency was reached.

AAV5 vectors were produced by triple transfection in HEK-293 cells and purified using cesium chloride (CsCl) gradient ultracentrifugation. To quantify AAV titers, Real-time quantitative polymerase chain reaction (RT-qPCR) was carried out in a 10μL volume in 96-well plates using Fast SYBR Green Master Mix (Applied Biosystems, Foster City, California, USA) by detecting the AAV sequence ITR. Primers for the ITR (forward: 5’- AAC ATG CTA CGC AGA GAG GGA GTG G -3’, reverse: 5’-CAT GAG ACA AGG AAC CCC TAG TGA TGG AG-3’) were designed and synthesized (Gene Script, New Jersey, USA). All RT-qPCRs included 40 cycles and a melt-curve. Serial dilutions of AAV with known titers were used as standards. Alkaline gel electrophoresis was also applied to verify AAV vector genome integrity; SYPRO Ruby protein gel stain (Thermo Fisher, Waltham, Massachusetts, US) was used to ensure the capsids contained all the three proteins, VP1, VP2 and VP3.

### Construction of AAV cassette for PD-L1 protein expression

Due to the homology of human PD-L1 and mouse PD-L1, human PD-L1 was reported to bind to mouse PD1 (28). Therefore, the human PD-L1 cDNA was synthesized and was cloned to pTR-CBh-GFP by substitution of the GFP transgene through the use of two restriction enzymes—Age I and NotI to generate pTR-CBh-PD-L1. The PD-L1 expression was driven by the CBh promoter that is able to induce a strong, long-term, and ubiquitous transgene expression (29). A 6* His-tag (CACCATCACCATCACCAT) was fused to PD-L1 right before the stop codon. The insert sequences were verified by Sanger sequencing, and the intact ITRs in the PD-L1 plasmid were verified by SmaI restriction enzyme digestion.

### Western blot

The pTR-CBh-PD-L1 plasmid was transfected into a 6 well plate of HEK-293 cells. After 48h, the cell medium supernatant was collected, and the HEK-293 cells were then lysed for 30 minutes on ice in RIPA buffer (Thermo Fisher, Waltham, Massachusetts, US) and x100 cocktail protein inhibitor (Thermo Fisher, Waltham, Massachusetts, US), followed by centrifugation at 13,000rpm for 10min at 4°C to separate the cell lysates from the cell debris. The supernatant or cell lysates were mixed with 4x Laemmli Sample Buffer (Bio-Rad), boiled for 7min, and separated by gel electrophoresis in 10% Mini-PROTEAN^®^ TGX™ Protein Gels precast gels (Bio-Rad Watford, UK). Recombinant human PD-L1 protein with His tag was used as a positive control (abcam, Cambridge, UK). After gel electrophoresis, proteins were transferred on nitrocellulose membranes (Bio-Rad) using a Trans-Blot Turbo Transfer System (Bio-Rad). Membranes were first blocked using blocking buffer (5% milk powder in TBS-T) and subsequently stained with mouse anti-6* His tag (abcam, Cambridge, UK) or mouse anti-human PD-L1 primary antibody (abcam, Cambridge, UK) overnight in antibody buffer (5% BSA in TBS-T). The blots were then washed in TBS-T, incubated with the HRP conjugated secondary antibody for 1h, and washed in TBS-T again, before visualizing with ECL substrate (GE Healthcare, Chicago, IL, USA) and imaging in an AI600 Chemiluminescent Imager (GE, Buckinghamshire, UK).

For detection of phosphorylated PI3K and AKT expression in activated T cells, T cells were lysed by RIPA lysis buffer and cocktail proteinase inhibitor (x100) at 4°C for 30min. The cell lysate was collected by centrifugation at 12,000rpm for 10 minutes at 4°C. 10 μg cell lysate from T cells were loaded in SDS-PAGE to detect p-PI3K and p-AKT protein by western blot using a rabbit anti- p-PI3K antibody (abcam, Cambridge, UK) and mouse anti- p-AKT antibody (abcam, Cambridge, UK). GAPDH was utilized as an internal control for tissue samples.

To verify the PD-L1 protein expression levels in the tissues, the mouse knees and the livers were collected and homogenized on dry ice, then incubated with T-PER™ Tissue Protein Extraction Reagent (Thermo Fisher, Waltham, Massachusetts, US) and cocktail proteinase inhibitor (x100) at 4°C for 30min. The supernatant was collected by centrifugation at 12,000rpm for 10 minutes at 4°C. 20 μg of supernatant from the tissue lysates were loaded in SDS-PAGE to detect PD-L1 protein by western blot using a mouse anti-6*His tag antibody (abcam, Cambridge, UK). GAPDH was utilized as an internal control for tissue samples.

### T cell functional assays

PD-L1 protein was purified from a PD-L1-transfected HEK-293 cells. The supernatant and cell lysates were collected and PD-L1 protein was purified using a HisTrap column (Cytiva, MA, USA); recombinant PD-L1 protein (R&D, MN, USA) was used as the positive control. Pan T cells from spleen were filtered through a 70 μm cell strainer (Miltenyi, Germany) and extracted by a T cell isolation kit (Miltenyi Biotec, Germany). T cells were cultured in RPMI medium 1640 with 2 mM L-Glutamine, 10% FBS, and 100 U/mL penicillin/streptomycin. T cells were stained with CellTrace Violet dye as indicated by the CellTrace™ Violet Cell Proliferation Kit (Thermo Fisher, Waltham, Massachusetts, US), then incubated with 2x10^5^ anti-CD3/CD28 beads (Thermo Fisher, Waltham, Massachusetts, US) and 10U/ml IL-2 (R&D, MN, USA) with or without 5 μg/ml of purified PD-L1. Recombinant PD-L1 and PBS were used as positive and negative controls, respectively. T cells cultured with no anti-CD3/CD28 beads were also designed. After 72h, T cells were collected, and the percentage of proliferating cells from each group were determined by Attune Flow Cytometer (Thermo Fisher, Waltham, Massachusetts, US) with an emission of 405/445nm. T cell apoptosis rate was also detected by anti-7AAD antibody (abcam, Cambridge, UK) with an emission of 550/650nm.

### Collagen induced arthritis mouse model

All animal care and housing requirements were followed under the guidance of the National Institutes of Health Committee on the Care and Use of Laboratory Animals of the Institute of Laboratory Animal Resources, and all animal protocols were reviewed and approved by the Institutional Animal Care and Use Committee at the University of North Carolina at Chapel Hill. The CIA model was used to mimic the acute inflammation conditions of RA. DBA/1J mice were selected due to the higher incidence and severity of RA manifestations compared to other strains such as C57BL/6 mice (30). Male DBA/1J mice at the age of 7-8 weeks were used.

For the primary immunization, bovine type II collagen (Chondrex, Woodinville, WA, USA) was dissolved with 0.01 N glacial acetic acid at a concentration of 2mg/ml, emulsified with complete Freund’s adjuvant (CFA) (Sigma-Aldrich, Burlington, MA, USA) using a “two-syringe” method for 1h on ice, and injected into the mouse (30). The booster vaccination was administered 21 days later with bovine type II collagen mixed with incomplete Freund’s adjuvant (IFA) (Sigma-Aldrich, Burlington, MA, USA). Collagen was injected into mouse tail root for both immunizations.

### Animal study design

For the prophylactic treatment, AAV5/PD-L1 vectors were injected into the knee joints on day 0 before arthritis was initiated, on the same day as the primary immunization of the type II collagen; We also administered AAV5/luc vectors or PBS into knee joints as control. For the therapeutic treatment, AAV5/PD-L1 vectors were injected on day 21, on the same day as booster vaccination, with the inflammation already gradually induced in joints. Mice received intra-articular administration of self-complementary (sc) AAV5/PD-L1 driven by the CBh promoter at a dose of 1x10^10^ particles in the left knee joint. From our pilot study, histology staining showed AAV5/luc didn’t enhance or decrease the joint inflammation in CIA mice model, and AAV5/luc didn’t trigger inflammation in naïve mice joints. There was no significant difference between AAV5/luc and PBS group in pathological score of joints from both CIA mice and naïve mice (**Figure S1**). Therefore, mice treated with PBS in the contralateral knee joints were used as control. For detecting the cytokine level in serum, the mice from untreated group received intra-articular administration of PBS in both knee joints. The negative control group consisted of naïve mice.

### Joint size measurement

The knee joint size (mm) from left to right side was measured using a caliper (31–33) (Mitutoyo, Takatsu-ku, Kawasaki, Kanagawa) at week 0 before primary immunization and weeks 3, 4, 5, 6, and 7 after primary immunization. The joint size increase percentage was calculated by the diameter of joints at different time points divided by the diameter of baseline size on day 0.

### Tissue histopathology

At week 7 after collagen primary immunization, mice were sacrificed and the knee joints were collected by dissecting the femur and tibia 5 mm away from the knee joint. The harvested knees were fixed in 4% paraformaldehyde, decalcified in 5% trichloroacetic acid for 7 days, dehydrated, embedded in paraffin, and sectioned at 5 μm for hematoxylin and eosin (H&E) staining and immunohistochemistry staining (IHC). Histopathological evaluation was conducted by two independent observers blinded to experimental group, and at least 10 fields of view per joint were analyzed. The score was based on following change in four conditions: synovial hyperplasia (0-3), infiltration of leukocytes into the synovial membrane/joint space (0-3), pannus formation (0-3), and necrosis/erosion of cartilage (0-3) (34).

Spleens and livers were fixed in 4% paraformaldehyde, dehydrated, embedded in paraffin, and sectioned at 5 μm for H&E staining.

To perform IHC, the sections were de-paraffinized, rehydrated, subjected to heat-induced antigen retrieval at 95°C for 20 min in 0.01 M sodium citrate, and cooled at room temperature for 25min. Subsequently, the samples were incubated in 3% H_2_O_2_ in methanol for 20 min, blocked with 10% normal goat serum for 1h, and incubated overnight at 4°C with following antibodies: rabbit anti-mouse CD68+ primary antibody (abcam, Cambridge, UK, 1/150 dilution) for macrophages, rabbit anti-mouse CD206 primary antibody (abcam, Cambridge, UK, 1/200 dilution), rabbit anti-mouse iNOS primary antibody (abcam, Cambridge, UK, 1/150 dilution), rabbit anti-mouse CD3+ primary antibody (abcam, Cambridge, UK, 1/150 dilution) for pan T cells, rabbit anti-IL-17 primary antibody (abcam, Cambridge, UK, 1/200 dilution), and rabbit anti-FOXP3 primary antibody (abcam, Cambridge, UK, 1/200 dilution), rabbit anti-6*His primary antibody (Thermo Fisher, Waltham, Massachusetts, US, 1/500 dilution), rabbit anti-murine PD-L1 antibody (abcam, Cambridge, UK, 1/500 dilution) and rabbit anti-human and murine PD-L1 antibody (Thermo Fisher, Waltham, Massachusetts, US, 1/1000 dilution) for PD-L1 expression. Negative controls were treated with 10% normal goat serum without the primary antibodies. Anti-rabbit antibody (abcam, Cambridge, UK, 1/500 dilution) was used as secondary antibody, the color was developed using a Vectastain Elite ABC Kit (Vector Laboratories, Burlingame, CA, USA) and DAB Substrate Kit (Vector Laboratories, Burlingame, CA, USA). 5 knee joints were used in each group, and 10 fields of view per joint were evaluated. Positively stained cells in each field of view were counted by two blinded observers and averaged.

### Cytokine assay

To examine the cytokines in the knee joint and serum at week 7 post primary immunization of collagen, the mice were sacrificed, knee joints were harvested, and the muscles were peeled off as much as possible. The bony structures were then minced and put into 500µl of cold PBS overnight in a 4°C cold room. The mouse blood was also collected by retro-orbital bleeding method using non-heparinized micro-hematocrit capillary tubes (DWK Life Sciences, Millville, NJ, US) and set for 30 min at room temperature. The supernatant from the joints and sera were then collected by centrifugation at 3,000rpm for 10 min. The protein concentration was measured by BCA assay. Multiple cytokines in the knee joint homogenization, including IL-1, IL-6, IL17A, TNFα, and IL-10, were measured on a Luminex MAGPIX system (Luminex Corporation, Austin, TX, USA). Cytokine levels were expressed in picograms per milliliter (pg/ml). Levels below the detection limit were defined as 0 pg/ml for each cytokine. The cytokine levels per mg protein were calculated.

### AAV copy number

DNA from the heart, liver, spleen, lung, kidney, and knee were extracted using DNeasy Blood & Tissue Kits (Qiagen, Germantown, Maryland, USA). AAV copy numbers in the heart, liver, spleen, lung, kidney, serum and knee were then determined by qPCR as described in our previous studies (35).

### Liver function

Aspartate aminotransferase (AST) and alanine aminotransferase (ALT) in serum at week 7 after primary immunization were measured at a wavelength of 340nm using AST/ALT Assay kit (abcam, Cambridge, UK).

### Detection of collagen antibodies

50ng/μl type II collagen were coated with 100 μl coating buffer (BioLegend, San Diego, CA, US) on Corning Costar Brand 96-Well EIA/RIA Plate (Thermo Fisher, Waltham, Massachusetts, US) overnight. After washing and blocking with 1% BSA buffer, mice sera or joint lysate (as mentioned in cytokine assay) were diluted from 1:10 to 1:107 with three times dilution and incubated for 2h at room temperature. After wash, HRP conjugated anti-mouse antibody (abcam, Cambridge, UK, 1/10000 dilution) was added in each well for 1h at room temperature, then TMB substrate (abcam, Cambridge, UK) was added for 15 min and reactions stopped with stop solution (Thermo Fisher, Waltham, Massachusetts, US), the color intensity was analyzed at 450nm, and the antibody titer was determined by the OD value that was 3 times higher than that in naïve mice.

### Statistical analysis

GraphPad Prism 9 software was used for statistical analysis. Data are shown as mean ± SD, and the box and whisker plots were used for descriptive statistics. Differences among each group were determined by one-way ANOVA or student’s t test. Bonferroni and Sidak tests were used for multiple comparisons between groups. The significance level was set at 0.05. Based on the power analysis of our preliminary data using nQuery software, the power of mouse sample size was over 80% at a significance level of 0.05.

## Results

### PD-L1 was expressed in HEK-293 cells

**Figure 1:**
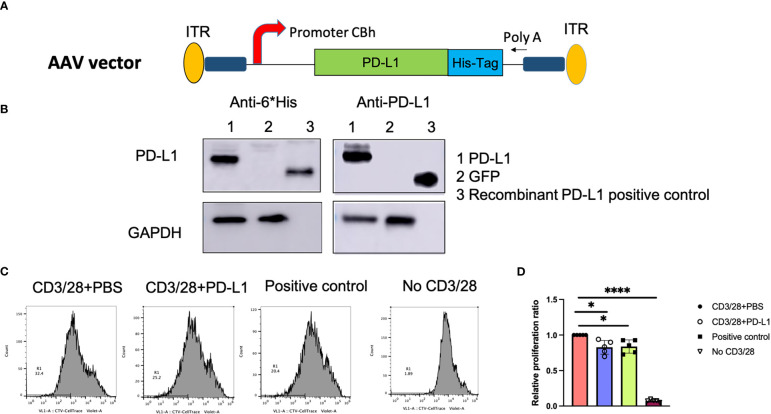
PD-L1 gene expression and function *in vitro*. **(A)**, Schematic diagram of the AAV/PD‐L1 cassette. **(B)**, Western blot analysis for PD‐L1 protein expression. After transfection of pTR/CBh-PD-L1 or pTR/CBh-GFP into HEK-293 cell lines, 48h later, the supernatant and cell lysate were collected for PD-L1 expression by western blot with antibodies against either His tag or PD-L1. Data shown is the representative of 5 independent experiments. **(C)**, T cell proliferation. Purified splenic T cells were stained with CellTrace Violet dye, then co-cultured with PBS, PD-L1 in the presence of anti-CD3/anti-CD28 for 72h. The proliferation of positively stained cells was analyzed with flow cytometry. Data shown is representative of 5 independent experiments. **(D)**, Summary data of T cell proliferation. The result was normalized to T cell proliferation rate in the group with CD3/28+PBS (n=5). Mean values are shown with standard derivation. Data was analyzed using one-way ANOVA followed by Bonferroni multiple comparison test for comparisons. *, p < 0.05, ****, p < 0.001.

To verify the human PD-L1 (hPD-L1) protein expression *in vitro*, we cloned the pTR-CBh-PD-L1 plasmid (**Figure 1A**), in which the hPD-L1 transgene is driven by the truncated CBA promoter, and transfected the plasmid in HEK-293 cells; both supernatants and cell lysates were then collected on day 2. PD-L1 was detected at 33kDa in cell lysates but not in supernatants. No PD-L1 was detected in HEK-293 cells transfected with pTR-CBh-GFP (**Figure 1B**).

### The effect of PD-L1 on T cell function inhibition

Next, PD-L1 protein was purified from transfected cells, and PD-L1 function was verified. Pan T cells were isolated and stained with CellTrace™ Violet, then activated and expanded with CD3/CD28 beads and IL-2, in the presence of PD-L1 protein. After 72h of T cell expansion, cells were resuspended in staining buffer and analyzed with flow cytometry. At the same threshold setting, the proliferation rates in the purified PD-L1 group and the positive control group (recombinant PD-L1 protein) were found to be similar without significant difference (25 ± 7.3% and 25.2 ± 6.5%, respectively; P>0.05). In contrast, the PBS group had a proliferation rate of 30.6 ± 9.9% (**Figures 1C, D**), and T cells without CD3/28 activation (inactivated cells) had a proliferation rate of 2.2 ± 0.6%. We normalized the proliferation rate in each group to that of the PBS group (**Figure 1D**) and found that PD-L1 from both the positive control and the pTR-CBh-PD-L1 transfection significantly decreased T cell proliferation(P<0.05). To further confirm the role of PD-L1 from our construct in inhibition of T cell function, we examined one of the downstream signals—PI3K-AKT pathway in T cells. We have tested expression of phosphorylated PI3K and AKT by western blot in the T cells incubated with PD-L1 from our construct, after 48h, we found less expression of p-PI3K and p-AKT when compared to T cells without treatment (**Figure S2**). Additionally, we also examined the apoptosis of activated T cells after 72h incubation by flow cytometry using anti-7AAD antibody. Higher apoptosis was found in T cells treated with PD-L1 when compared to PBS (**Figure S2**). These results clearly support that PD-L1 from our construct is able to block activated T cell function.

### PD-L1 expression in AAV5/PD-L1 transduced mouse knee joint

**Figure 2:**
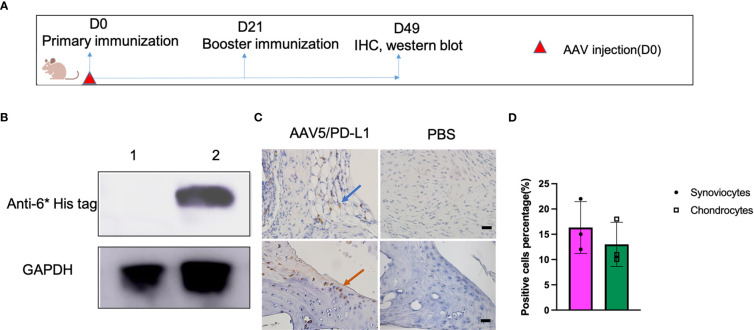
High PD-L1 expression in knee joints treated with AAV5/PD-L1 vectors. **(A)**, Schematic diagram of animal experiment. On day 0, AAV5/PD-L1 vectors at a dose of 1x10^10^ particles were injected in CIA mouse model into the left knee, and PBS into the right knee of the same CIA mice. On the same day, type II collagen was injected into the mouse tail root as the primary immunization, followed by booster immunization on day 21. Joints were collected for PD-L1 detection on day 49. **(B)**, Western blot of PD-L1 expression in knee. Knee joints from both left knee (AAV5/PD-L1 injection) and right knee (PBS) were lysed and the supernatant was collected for PD-L1 protein detection by western blot using mouse anti-6*His tag antibody. Data shown is representative of 3 independent experiments. **(C)**, Immunohistochemistry staining for cells transduced by intra-articular injection of AAV5/PD-L1 vectors. Representative images are shown with anti-6* His positive cells in AAV5/PD-L1 treated mouse knees (left) and PBS treated mouse knees (right) (n=3, bar=20μm). The blue arrow indicated positively stained synoviocytes, the orange arrow indicated positively stained chondrocytes. **(D)**, PD-L1 expression cell percentage in AAV5/PD-L1 treated joints. Three knee joints were analyzed in each group, the positive PD-L1 cells were counted from 10 fields of view and averaged. Each dot represents one mouse joint. The data is represented by mean and standard derivation. Data was analyzed by two-tailed paired student’s t test.

To examine PD-L1 expression *in vivo*, AAV5/PD-L1 vector was injected into the left knee, and PBS was injected into the right knee of the same CIA mouse as a control (**Figure 2A**). At week 7 post AAV injection, we collected mouse knee joints and carried out a western blot to detect PD-L1 expression in knee joint lysates. PD-L1 was detected in the knee joint lysates of the AAV5/PD-L1 injected group, but not in the PBS group (**Figure 2B**). PD-L1 expression was further validated by IHC staining with anti-6* His tag. In AAV5/PD-L1 injected mice, approximately 15% of synoviocytes and 12% chodrocytes were detected as positive in the AAV5/PD-L1 group (**Figures 2C, D**) but not in PBS injected group. These findings indicate that locally injecting AAV/PDL1 vector into the joints induced PD-L1 expression.

### Prophylactic local injection of AAV5/PD-L1 vectors reduced knee joint swelling and clinical scores in RA mice

**Figure 3:**
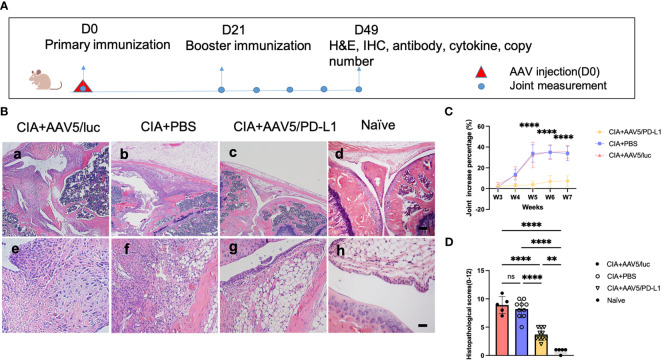
Improved knee joint swelling and decreased histological change in joints treated with AAV5/PD-L1 vectors as prophylaxis. **(A)**, Schematic diagram of animal experiment with intra-articular injection of AAV5/PD-L1 vectors as a prophylactic treatment, 1x10^10^ particles of AAV5/PD-L1 vectors were injected into the left knees of CIA mice on day 0, while PBS or AAV5/luc was injected into the right knees of the same mice. On the same day, the primary immunization with type II collagen was applied, and on day 21, the booster immunization was applied. Knee joint size was measured weekly. At week 7, joints were collected for histology and cytokine analysis, and other tissues, including sera, liver, spleen, heart, lung, and kidney, were also harvested to evaluate systemic impact of PD-L1 expressed in the joints. **(B)**, joint histology analysis with H&E staining. Representative images of H&E staining from the CIA mouse knees injected with PBS, CIA mouse knees injected with AAV5/PD-L1, AAV5/luc or naïve mice at week 7 are shown (n=10, a-d, bar=200μm; e-h, bar=20μm). **(C)**, knee joint swelling change with PD-L1 prophylactic treatment. The knee joint sizes at different time points were measured and compared to those on day 0. Data is shown as means ± SEM (n=10). ****, p < 0.001. ​ **(D)**, The knee joint histological score (n=10). Histopathological evaluation was performed and scored by two independent observers for the following changes: synovial hyperplasia, leukocyte infiltration, pannus formation, and cartilage necrosis/erosion. Each dot represents one mouse joint. Data is represented as means ± SEM. Data was analyzed using one-way ANOVA followed by Bonferroni multiple comparison test for group comparisons. **, p < 0.01, ****, p < 0.001. ns, not significant (p ≥ 0.05).

To investigate whether the local PD-L1 expression from AAV vector gene therapy was able to prevent knee joint inflammation, the well-established CIA mouse model was used. DBA/1J mice were treated with AAV5/PD-L1 vectors *via* intra-articular injection of 1x10^10^ particles in the left joint and PBS in the right joint on day 0. On the same day, type II collagen was injected into the tail root (**Figure 3A**). The booster of type II collagen was applied on day 21. At that time, the mice were already exhibiting some degree of swelling around the joint. The size of the joints with PBS and AAV5/luc treatment gradually increased to their peak by day 35 post primary collagen immunization and remained unchanged over the next two weeks. Compared to their size before primary collagen immunization, the size of joints injected with PBS had increased by 33.8 ± 6.9% and joints injected with AAV5/luc had increased by 35.43 ± 6.2% (**Figure 3C**). However, the joint swelling of the AAV5/PD-L1 treated group reached the peak one week earlier than that of the PBS group, and joint size had only increased by 6.2 ± 3.9% on day 49 post AAV intra-articular injection, significantly reduced as compared to the PBS group in CIA mice (P<0.05).

At week 7, the mice were sacrificed. Knees were collected and sectioned for H&E staining, and the histopathological change was graded based on synovial hyperplasia, immune cell infiltration, pannus formation, and cartilage necrosis/erosion (**Figure 3B**). As shown in **Figures 3B, D**, AAV5/PD-L1-treated knees had a significantly lower histopathological score compared to that of the PBS and AAV5/luc group (P<0.05); for comparison, the histopathological score of the untreated knees of naïve mice was 0.8± 0.4(P<0.05) (**Figure 3D**). These results implicate the prophylactic effect of PD-L1 expressed from AAV transduced synoviocytes on arthritis development in CIA mice.

### PD-L1 expression decreased T cell infiltration in the knee

**Figure 4:**
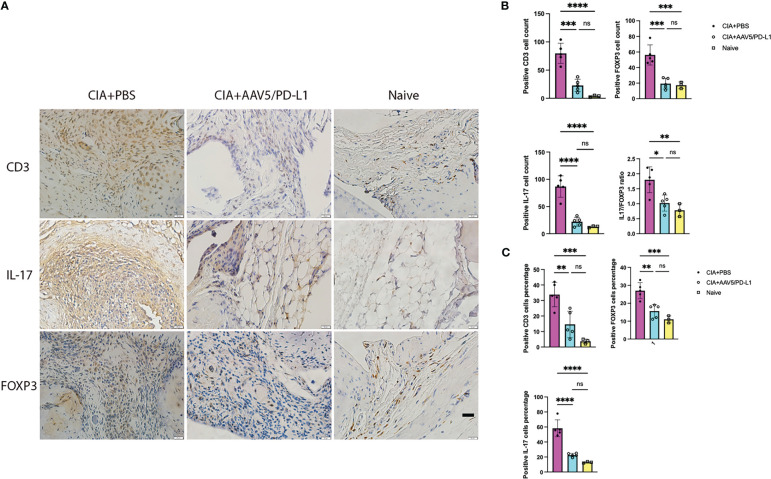
Infiltration of T-cells in the synovium was reduced in PD-L1 treated joints with AAV vectors for prophylactic treatment. **(A)**, Immunohistochemistry staining for T cells. Representative images of *in situ* immunohistochemical visualization of T cells stained with antibodies against CD3, IL-17, FOXP3 (bar=20μm) is shown in CIA mouse knees treated with PBS, AAV5/PDL1, or naïve mice. B-C, Total number **(B)** and cell percentage **(C)** for CD3, IL-17 and FOXP3. The cells were counted in 10 fields of view and averaged in each joint. Each dot represents one mouse joint. Five knee joints were analyzed in each group. Mean values are shown with standard derivation (n=5). Data was analyzed using one-way ANOVA followed by Bonferroni multiple comparison test for group comparisons. *, p < 0.05, **, p < 0.01, ***, p < 0.005, ****, p < 0.001. ns, not significant (p ≥ 0.05).

T cells play a critical role in the initiation and progression of RA. T cells were stained with a CD3 antibody with brown color (**Figure 4A**) and T cells infiltrating the synovium of each joint were counted and averaged in 10 fields of view (x400) under the microscope. There was a remarkable decrease of T cell infiltration in AAV5/PD-L1 treated joints compared to those of the PBS group (P<0.05). The T cell numbers were 79.8 ± 17.8, 22.8 ± 10.8 and 3.7 ± 1.5 in the synovium of the CIA+PBS, CIA+AAV5/PD-L1, and naïve joints, respectively (**Figure 4B**). We also further analyzed cells expressing IL-17 or FOXP3 (**Figure 4A**), and found that positive IL17 cells were ~4 fold higher in the CIA+PBS group than that in the CIA+AAV5/PD-L1 group (**Figure 4B**, 86.8 ± 19.7 vs 22 ± 8.2, P<0.05). Positive FOXP 3 cells were ~2.5 fold higher in CIA+PBS group than CIA+AAV5/PD-L1 group (**Figure 4B**, 56.9 ± 12.9 vs 19.4 ± 7, P<0.05). The same trend in positive cell percentages was observed as cell numbers (**Figure 4C**). The ratio of IL17+ cells to FOXP3+ cells was 1.8, 1.0, and 0.7 in the PBS, AAV5/PD-L1, and naïve groups, respectively. The ratio of IL17/FOXP3 was significantly higher in the PBS group than that in both the AAV5/PD-L1 group and the naïve group (P<0.05), but no significant difference was observed between the AAV5/PD-L1 group and the naïve group (P>0.05, **Figure 4B**).

### PD-L1 expression decreased macrophage infiltration in the knee

**Figure 5:**
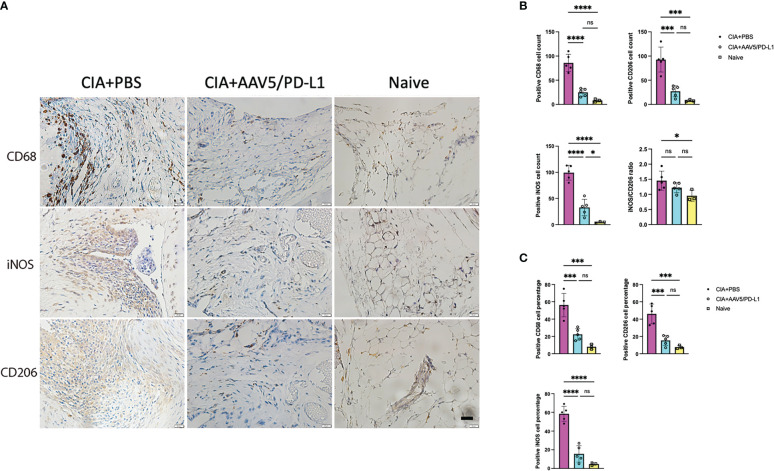
Decreased infiltration of macrophages in the synovium with PD-L1 prophylactic treatment. **(A)**, Immunohistochemistry staining for macrophage cells. Representative images of *in situ* immunohistochemical visualization of macrophages stained with antibodies against CD68, iNOS, CD206 (bar=20μm) are shown in CIA mouse knees. **(B, C)**, Total number **(B)** and cell percentage **(C)** for CD68, iNOS and CD206. The macrophages were counted from 10 fields of view and averaged in each joint for five knee joints. Each dot represents one mouse joint. Mean values are shown with standard derivation (n=5: PBS and AAV5/PD-L1; n=3: naïve). Data was analyzed using one-way ANOVA followed by Bonferroni multiple comparison test for group comparisons. *, p < 0.05, ***, p < 0.005, ****, p < 0.001. ns, not significant (p ≥ 0.05).

To verify if PD-L1 decreased macrophage infiltration in mouse knees in the CIA model, macrophages were stained with CD68 antibody (**Figure 5A**), and the infiltration of macrophages in the synovium was quantified. The macrophage number in the PBS group was found to be ~5.5 fold higher than that of the AAV5/PD-L1 treated joints (P<0.05) and ~20 fold higher than that of the naïve mice (**Figure 5B**, 85.8 ± 18, 15.6 ± 8.8, and 4.7 ± 1.5 respectively). Next, we investigated the infiltration of different subsets of macrophages using iNOS staining for M1 macrophages and CD206 antibody staining for M2 macrophages. As shown in **Figure 5B**, the number of iNOS+ cells in the PBS group was ~3 fold higher than that of the AAV5/PD-L1 group (99.4 ± 14.3 vs 32.8 ± 15.7, P<0.05). Similarly, about 3 fold more CD206+ cells were detected in the PBS group than in the AAV5/PD-L1 group (**Figure 5B**, 92.6 ± 25.3 vs 27 ± 11.5, P<0.05). There was a significant difference in the percentage of total macrophages as well as cells expressing iNOS or CD206 between the AAV5/PD-L1 group and PBS group, but no significant difference in cell percentages between AAV5/PD-L1 group and naïve mice (**Figure 5C**). Furthermore, we calculated the iNOS+/CD206+ ratio by dividing the positive iNOS cell number by the positive CD206 cell number, and found that the ratios were ~1.5, 1.2, and 0.9 in the PBS, AAV5/PD-L1 and naïve groups, respectively; there was no significant difference between the AAV5/PD-L1 group and the PBS group or between the AAV5/PD-L1 group and the naïve group(P>0.05, **Figure 5B**). However, a significant difference was found between the PBS group and the naïve group (P<0.05, **Figure 5B**).

### AAV5/PD-L1 decreased inflammatory cytokine levels in the knee

**Figure 6:**
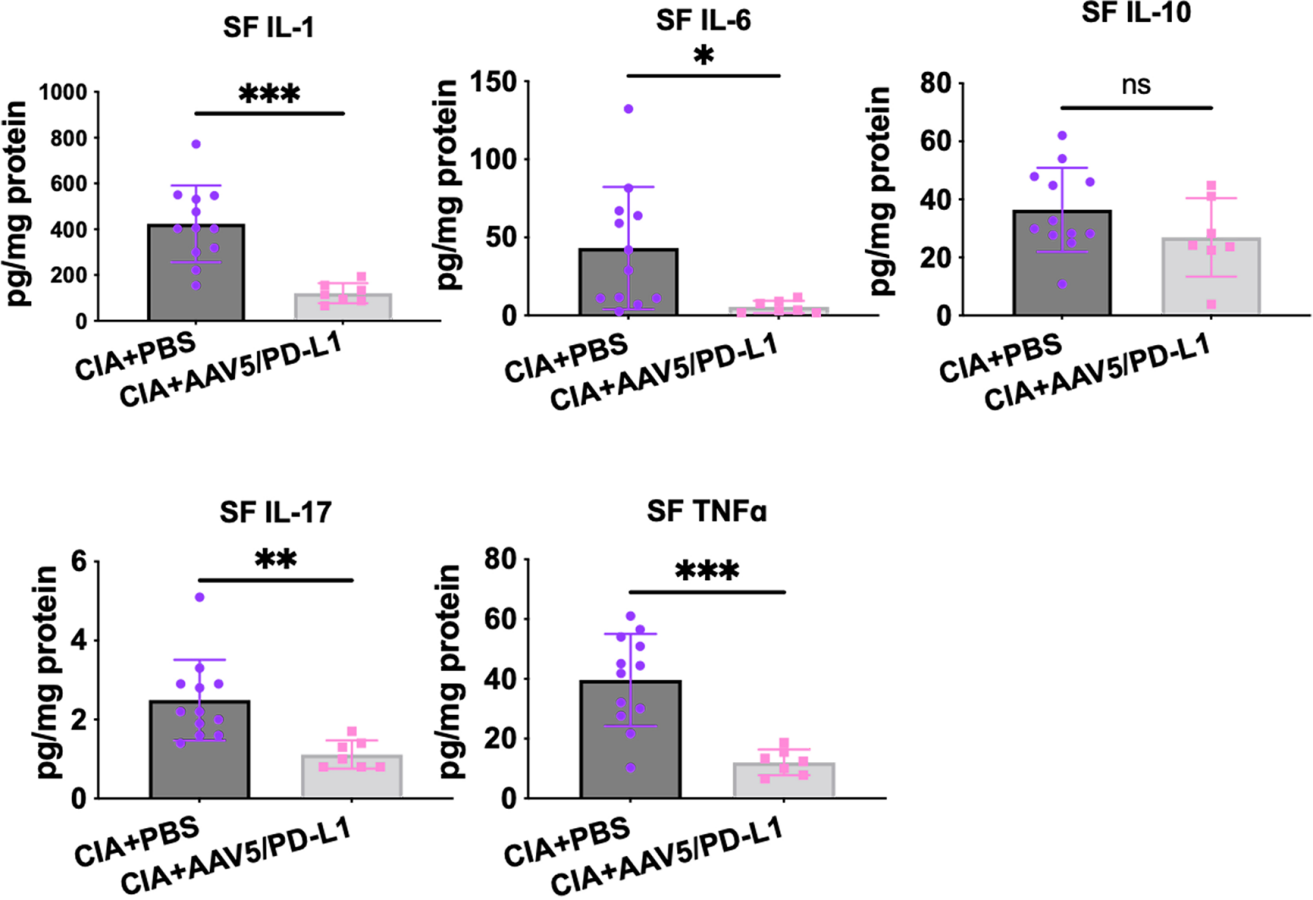
Reduced cytokines level in knee joints treated with AAV5/PD-L1 vectors. Knee joints were homogenized and the supernatant was collected for detection of cytokines IL-1, IL-6, IL17A, TNFα, and IL-10. Mean values are shown with standard derivation (n = 12: PBS; n = 7: AAV5/PD-L1). Data was analyzed using two-tailed unpaired student’s t test. *P < 0.05, **P < 0.01, ***, p < 0.005. ​ SF, synovial fluid. ns, not significant (p ≥ 0.05).

Immune cell infiltration and activation induces production of proinflammatory cytokines/chemokines. We studied whether PD-L1 treatment in the joints impacted the production of cytokines. Five representative cytokines were analyzed in local joint lysates by a Luminex MAGPIX system. As shown in **Figure 6**, the levels of pro-inflammatory cytokines IL-1, IL-6, IL-17, and TNFα in the AAV5/PD-L1 treated knee joint tissue homogenization were 2~5 fold lower than those in the PBS groups (P<0.05), while the level of inhibitory cytokine IL-10 was not different between AAV5/PD-L1 and PBS groups (P>0.05). The cytokine levels in the knee joints of naïve mice were undetectable (data not shown). This result showed that local AAV5/PD-L1 treatment decreased the generation of proinflammatory cytokines in the knee joints of CIA mice.

### AAV5/PD-L1 decreased mouse endogenous PD-L1 expression in the knee

It has been demonstrated that inflammation could up-regulate PD-L1 expression. For example, IFN-γ, IL-2, IL-17, IL-15, IL-4 and granulocyte-macrophage colony-stimulating factor (GM-CSF) have been considered potent inducers of PD-L1 expression (36, 37). Therefore, we have performed the immunohistochemistry staining to detect PD-L1 expression in joints of CIA mice. When anti-murine PD-L1 antibody with cross-reactivity to human PD-L1 was used, total cells expressing PD-L1 were similar in joints regardless of PD-L1 treatment. However, when we applied the antibody only detecting mouse PD-L1, we found a decrease in mouse PD-L1 expression in cells in the joints treated with AAV5/PD-L1 vectors (**Figure S3**). The up-regulation of PD-L1 expression upon inflammation in arthritis is perhaps caused by the compensation feedback of host response to inflammation and then attenuates the disease. Collectively, these results indicate that early delivery of AAV vectors for PD-L1 expression could decrease joint inflammation and then reduce mouse endogenous PD-L1 expression in joints with arthritis.

### Intra-articular injection of AAV5/PD-L1 vectors did not induce PD-L1 expression in other tissues

**Figure 7:**
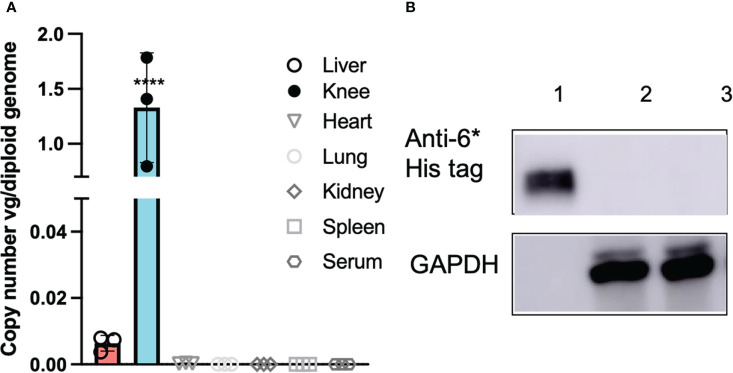
No systemic side effects detected after intraarticular AAV5/PD-L1 injection. **(A)**, AAV genome copy numbers. At week 7 after AAV injection, mice were euthanized and different tissues (liver, knee, heart, lung, spleen and kidney) were collected for AAV genome analysis using qPCR. DNA from the heart, liver, spleen, lung, kidney, and knees from mice injected with AAV5/PD-L1 were extracted using DNeasy Blood & Tissue Kits and then determined by qPCR. The data represent the mean and standard derivation (n=3). ​****, p < 0.001. **(B)**, Western blot of PD-L1 in liver lysates. 20 μg of supernatant from the liver lysates collected from mice injected with AAV5/PD-L1 and PBS were loaded in SDS-PAGE to detect PD-L1 protein by western blot using mouse anti-6*His tag antibody. Lane 1, recombinant PD-L1-his protein, lane 2, AAV5/PD-L1 treated mice liver, lane 3, PBS treated mice liver.

Ideally, local PD-L1 application would induce minimal effects on systemic immune responses. In order to elucidate whether intra-articular injection of AAV5/PD-L1 vectors induce a significant systemic immune response modulation, we first studied AAV genome biodistribution using qPCR in different tissues including joints, liver, spleen, kidney, heart and serum. Besides high AAV genomes in the AAV5/PD-L1 treated knees with ~ 1.3 viral copy numbers per diploid genome, AAV genomes were also detected in the liver at levels of about 0.008 viral copy numbers per diploid genome, approximately 150-fold lower than that of the joints (P<0.05). AAV genomes were undetectable in other tissues with less than 0.001 copy per diploid genome (**Figure 7A**). We further checked PD-L1 expression and found no detectable PD-L1 in the livers of CIA mice after intra-articular administration of AAV5/PD-L1 vectors (**Figure 7B**).

### Intra-articular AAV5/PD-L1 treatment did not change cytokine levels in serum

**Figure 8:**
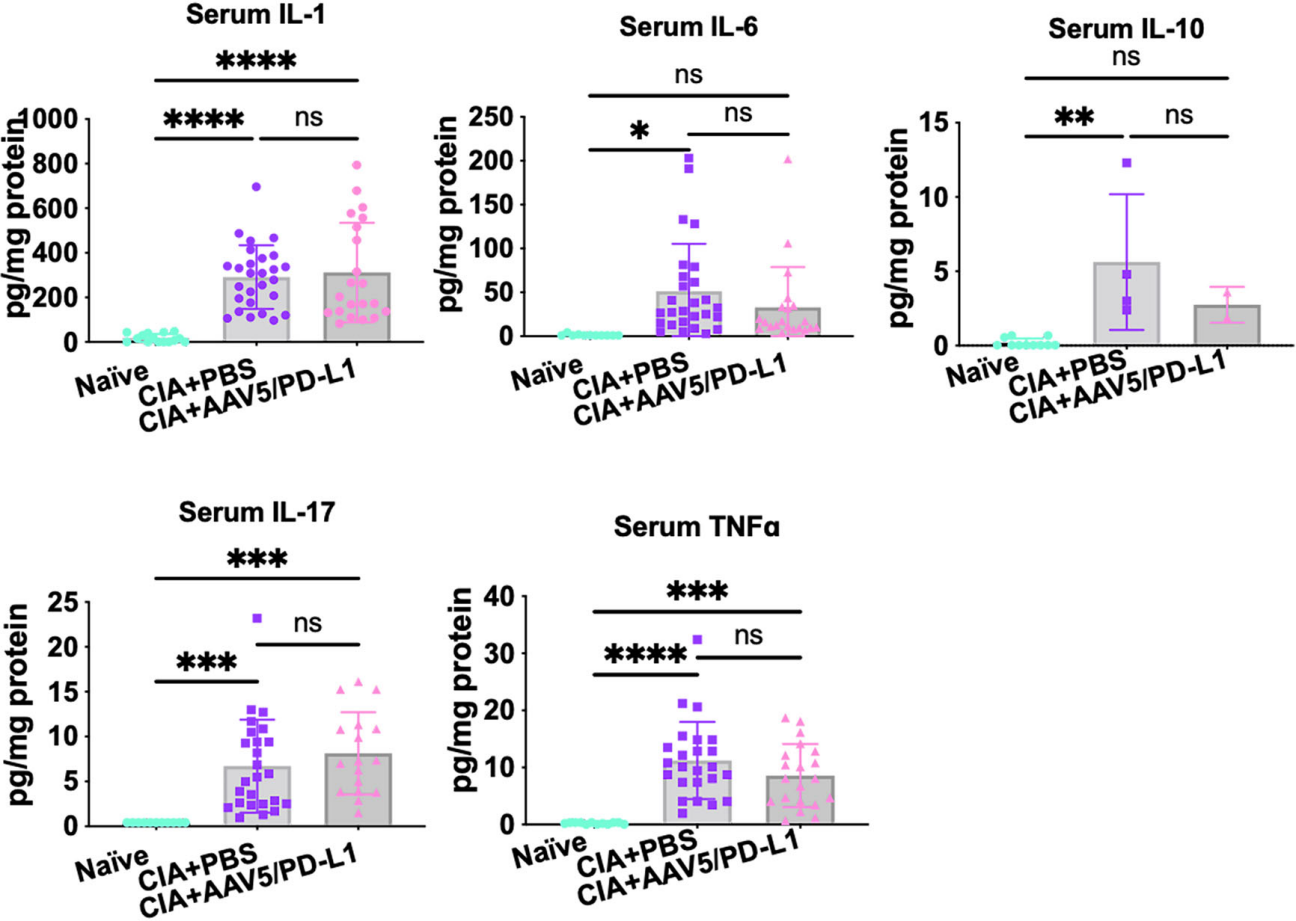
The levels of multiple cytokines in mouse serum. Mouse sera were collected from both CIA mice that had been injected with or without AAV5/PD-L1. The concentrations for IL-1, IL-6, IL17A, TNFα, and IL-10 were measured. The mean values are shown with standard derivations. *P < 0.05, **P < 0.01, ***, p < 0.005, ​****, p < 0.001. ns, not significant (p ≥ 0.05).

To determine if joint PD-L1 treatment had a similar effect on cytokine levels in serum as in the joints, we further investigated cytokine levels in the blood serum from CIA mice treated with AAV5/PD-L1 or PBS, as well as in naïve mice. Higher cytokine levels were detected in CIA mice than in naïve mouse serum (P<0.05) (**Figure 8**), but there was no significant difference in serum cytokine levels of CIA mice regardless of treatment with AAV5/PD-L1 vectors or not (P>0.05). This result implies that intra-articular AAV5/PD-L1 treatment does not have an impact on the systemic cytokine profile in sera of CIA mice.

### Intra-articular administration of AAV5/PD-L1 vectors did not impact the antibody titers against type II collagen

The CIA model was established by immunization of two doses of type II collagen antigen. We studied whether the local administration of AAV5/PD-L1 vectors influences the collagen specific antibody titers in sera and joints. In both the serum and knee joint lysate, similarly high titers of antibodies against type II collagen were generated in CIA mice treated with AAV5/PD-L1 and PBS, with a type II collagen antibody titer of ~3.3x10^5^ per μl in serum and ~3.3 x10^4^ per 1mg in knee joint lysate, but not in naïve mice (**Table 1**). These results suggest that local joint PD-L1 expression is unable to affect antibody production and local specific antibody titers in CIA mice.

**Table 1:** Antibody titer against type II collagen in mouse serum and knee.

	CIA+PBS	CIA+AAV5/PD-L1	Naïve
**Antibody** **titer in serum**	**3.3e5**	**3.3e5**	**< 10**
**Antibody titer in knee/mg protein**	**3.3 e4**	**3.3 e4**	**< 10**

### Local joint PD-L1 expression did not impact general health conditions in CIA mice

After immunization with type II collagen, CIA mice manifested redness and swelling in paws without mobility change. After intra-articular administration of AAV5/PD-L1 vectors, we only observed a slight increase in body weight during the time of observation, without significant differences among groups (**Figure S4**). No death from the PD-L1 treatment was observed.

We also analyzed histological changes in the liver and spleen. Hepatocytes and splenocytes were normal in shape and arrangement with no macrovesicular steatosis, necrosis, or apoptosis, indicating no signs of inflammation, cellular damage, or carcinoma (**Figure S5**). At week 7 after AAV administration, the levels of AST and ALT in sera were within normal range in all mice, regardless if treated with AAV5/PD-L1 or not (**Figure S6**).

### Local joint injection of AAV5/PD-L1 vectors blocked the progression of arthritis in CIA mice

**Figure 9:**
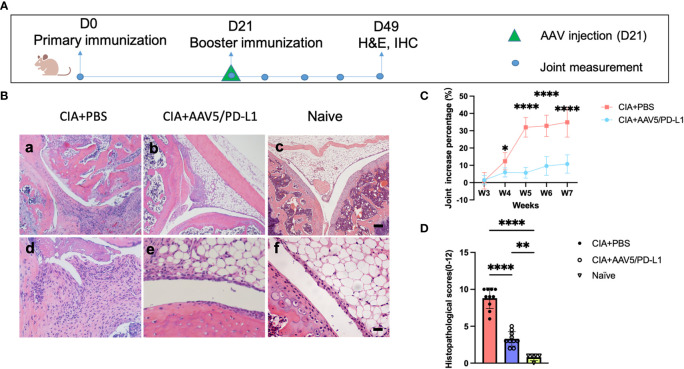
Improved arthritis in the synovium of CIA mice treated with AAV5/PD-L1 vectors as a therapeutic regimen. **(A)**, Schematic diagram of animal experiment. AAV5/PD-L1 at a dose of 1x10^10^ particles was intra-articularly injected on day 21 into the knees of CIA mice. Knee joint size and joint histopathological alteration were monitored. **(B)**, Knee joint histology analysis with H&E staining. Representative images are shown (a-c, bar=200μm; d-f, bar=20μm). **(C)**, The change in knee joint swelling. Data is shown as means ± SEM. *P < 0.05, ​****, p < 0.001. **(D)**, Knee joint histological scores. Mean values are shown with standard derivation (n=10). Data was analyzed using one-way ANOVA followed by Bonferroni multiple comparison test for group comparisons. **P < 0.01, ​****, p < 0.001.

As shown above, we had demonstrated that administration of AAV5/PD-L1 was able to prevent arthritis development in the CIA model. Next, we addressed whether PD-L1 expressed from AAV transduced joints had the potential to block the progression of arthritis, since RA is a chronic disorder with intermittent remissions and flare ups. To execute the study, AAV5/PD-L1 vectors were injected in the left knee on day 21 post primary immunization of type II collagen in CIA mice instead of on day 0 (**Figure 9A**). Consistent with the results of AAV5/PD-L1 vectors injected on day 0, mice treated with intra-articular injection of AAV5/PD-L1 vectors on day 21 developed a ~4-fold reduction of swelling of the knee joint compared to the untreated knee joints by week 7 (**Figure 9C**, 10.8 ± 5.3 vs 34.9 ± 8.6, P<0.05), as well as a ~2.5-fold decrease in histopathological scores (**Figures 9B, D**, 3.3 ± 1 vs 8.8 ± 1.4, P<0.05). Again, the infiltration of T cells and macrophages experienced a 2~4-fold decrease in joints treated with AAV5/PD-L1 compared to those treated with PBS (**Figure S7**).

## Discussion

RA is a chronic inflammatory disease primarily involving the joints. Mainstream therapies have been aimed at reducing synovial inflammation and pain, as well as preventing joint destruction by targeting pro-inflammatory cytokines or immune cells including B cells and T cells. However, over 50% of patients lack adequate responses to current available immunomodulatory therapies. In this report, we investigated a novel gene therapy approach using AAV vectors to deliver PD-L1 into the knee joints, and demonstrated that a one-time intra-articular injection of AAV5/PD-L1 vectors was able to decrease knee joint swelling, block the infiltration of lymphocytes and macrophages, and decrease pro-inflammatory cytokines in joints without impacting systemic immune responses. The PD-L1 expressed from direct injection of AAV vectors into the joints was able to prevent both the development and progression of arthritis in CIA mice.

The broad impact of PD-L1 on immune cells suggests that the PD-1/PD-L1 pathway may be involved in multiple autoimmune diseases and so it has been explored for therapeutic purposes. Nasr et al. reported that the overexpression of PD-L1 in autologous hematopoietic stem and progenitor cells (HSPCs) reversed hyperglycemia in 100% of NOD mice as a model of type 1 diabetes, with long-term effects observed in ∼30% of mice (38). Similarly, Zhang, et al. showed that engineered PD-L1-expressing platelets were able to reverse type 1 diabetes (T1DM) (39). There is evidence that PD-L1 heterogeneity is directly related to RA in some patients. The downregulation of PD-1 inhibitory signaling in RA might be due to increased levels of PD-1 in serum and decreased levels in synovial tissue, leading to fewer interactions between PD-L1 and PD-1 in RA synovial tissue (27). Additionally, the availability of PD-L1 could be limited due to increased expression of CD80 and the binding of CD80 to PD-L1 in cis, therefore reducing the amount of functionally available PD-L1 able to access PD-1 on T cells (40). These data all highlight that PD-L1 should be considered as a potential treatment target for a wide variety of autoimmune disorders (27).

Besides T cells, other immune cells, including macrophages, NK cells, B cells, and antigen presenting cells also express the PD-1 molecule (41). It is well known that immune cells such as T cells and macrophages infiltrating joints play a major role in pathogenesis of arthritis. Theoretically, targeting these immune cells would reduce joint inflammation (42). After treatment with AAV5/PD-L1 vectors, total T cells and macrophages as a whole, along with IL17+cells, CD206+cells, FOXP3+cells and iNOS+ cells, were all reduced in joint tissues. This result is consistent with findings that mice with CD4+ T cell or macrophage deficiencies developed less arthritis induced by collagen immunization (43, 44). In the present study, the ratio of IL-17+ cells/FOXP3+ cells in PD-L1 treated joints also decreased compared to control; however, the ratio of iNOS+/CD206+ cells for the AAV5/PD-L1 treated group was similar to that of the group without PD-L1 treatment. These findings may indicate that the effect of PD-L1 treatment is mainly mediated by blocking total immune cell infiltration and shifting proinflammatory T cells to anti-inflammatory T cells, but not by shifting macrophages from proinflammatory to anti-inflammation states.

A current focus in RA therapy is cytokine inhibition, including TNF-α (45), IL-6 (46), and IL-1 (47). However, TNF-α inhibitors showed approximately 30-50% efficacy in clinical reports, with less than 50% of patients showing alleviation (6). Similarly, high doses of the IL-6 inhibitor tocilizumab showed only 20% improvement in ~50% patients (46) and expression of an IL-1R antagonism showed only a mild response, possibly due to other pathways such as synovial TLR ligands that bypass IL-1-dependent signaling (48). Recently, one clinical trial reported that IFN-β provided *via* AAV gene delivery was insufficient for patients and the trial was paused (49).

Compared to published results from targeting single cytokines, our approach of targeting an immune checkpoint pathway showed much greater promise. An improvement of approximately 75% was achieved in histopathology and clinical manifestations, with almost every mouse having some degree of positive response after AAV5/PD-L1 treatment. However, a complete response without joint swelling and histopathological change was not demonstrated. There are several possible reasons why our AAV-PD-L1 therapy did not result in complete recovery from inflammation. First, systemic proinflammatory cytokines may play a role in the pathogenesis of arthritis in RA. Local PD-L1 expression is capable of inhibiting immune cells from secreting cytokines in the joint but are unable to block cytokines in the blood that could enter the joints. This can also partially explain why proinflammatory cytokines were still detected in PD-L1 treated joints even though the immune cells had reduced to the level in joints of naïve mice. Second, as the dose of AAV vectors we used in this study was not optimal, increasing the AAV vector dose may transduce more cells in the joint and improve the PD-L1 effect. In this study, only 15% of cells in the joints were transduced when 1x10^10^ particles of AAV5 vectors were administered. Third, collagen specific antibodies circulate in the blood and will ultimately penetrate the joint barrier and enter the joint synovia to interact with joint synoviocytes, inducing joint inflammation and damage. Although local joint PD-L1 expression decreased the level of immune cells in the joints, it did not block antibody production; as shown in our result, similar levels of antibodies against collagen were found in both the blood and joints of CIA mice regardless of whether PD-L1 treatment was applied or not.

To enhance PD-L1 function, several strategies can be explored. First, AAV vector modification, including capsid engineering, optimized PD-L1 sequence, or the development of stronger promoters, can be used to enhance AAV transduction (50). Second, AAV vectors encoding soluble PD-L1 can be designed, which allows PD-L1 to enter the joint fluid broadly without being restricted by cell type, as AAV vectors encoding wild type PD-L1 with a transmembrane domain can only be expressed on a limited array of cells. Lastly, PD-L1 could be combined with other approaches to block antibodies in joints to enhance therapeutic effects, such as immunoglobulin cleavage enzymes IdeS or IdeZ (51).

Local joint gene delivery of therapeutic proteins has been actively explored for RA in clinical trials in recent years. A limitation of intra-articular therapy for RA is that only one or a few joints could be practically treated. However, a recent study demonstrated that joint inflammation in RA patients tends to recur in the same joints over time (52) and so those specific joints could be targeted. Additionally, children with certain forms of inflammatory arthritis may only have a mono or oligoarticular synovitis that could be amendable with IA therapy (53). An advantage to the present approach is that AAV transduction is able to induce persistent transgene expression and so intra-articular administration of AAV5/PD-L1 is a feasible strategy and will likely benefit RA patients in the long term.

Overexpression of PD-L1 in other tissues could cause unwanted side effects. For example, PD-L1 in the liver might cause immune suppression, which could potentially facilitate infections and even liver tumorigenesis. To decrease the systemic impacts, AAV5 was used as the viral vector to deliver PD-L1, as this serotype showed a good transduction efficiency in the knee joint, transduced both synoviocytes and chondrocytes (54, 55), and demonstrated limited liver transduction (54, 55) when compared to other serotypes of AAV. Consistent with the results from previous reports using AAV5 (56), PD-L1 expression was exclusively detected in AAV5 vector-transduced joints. Even though PD-L1 effectively blocked immune cell infiltration and decreased the cytokines in the knee joint, it didn’t impact systemic immunity, because the cytokine levels and collagen antibody titers in serum were not altered. We also see the swollen paws and high arthritis scores in untreated joint of one leg, but low arthritis scores in the AAV/PD-L1 treated knees with the swollen paws of another leg. This further supports that the therapeutic effect of AAV5/PD-L1 injection was confined within the local joints. In addition, mice treated with AAV5/PD-L1 in joints didn’t show any abnormalities in other tissues, such as liver disfunction. These results all indicate a good safety profile of intra-articular injection of AAV5/PD-L1 vectors for arthritis treatment.

In this study, both prophylaxis and therapeutic treatment regimens were investigated to mimic two different scenarios: gene therapy before RA development and during early phase of RA. For the prophylactic treatment, instead of injecting AAV after the development of acute inflammation, AAV was injected at the same time as CIA induction. This decision was related to the characteristics of RA as a chronic autoimmune disease with both flare-ups and remissions; if the patient is able to get preventative treatment during remission, it may prevent them from reexperiencing acute inflammation. For the therapeutic treatment, AAV was injected after CIA induction, the rationale being that most patients will seek long-term treatment only after symptoms are developed or during the progression. Though persistent expression of PD-L1 after AAV delivery is favorable for the treatment of a chronic disease like RA, there is still concern about overexpression of PD-L1 causing long-term immune response suppression once the arthritis is in remission. However, we have followed up with wild type mice injected with AAV5/PD-L1 into the knees for over 1 year, and have not observed any significant adverse events (data not shown). This may be attributed to the knee joint anatomy, which is an enclosed environment with very few exposures to bacteria and other microbes; this may help in avoiding infection even when local immunity is suppressed. For future study, in order to regulate PD-L1 expression in different inflammatory conditions and avoid side effects related from long-term overexpression of PD-L1, inflammation inducible promoters might be considered to regulate transgene expression in matching disease activity.

In conclusion, this study laid a foundation for potential clinical applications of PD-L1-based therapies. PD-L1 have successfully attenuated inflammation and improved the pathology with decreased lymph cells and cytokine levels in the CIA joints. The precise mechanisms of PD-L1 need to be further investigated. It is promising that one-time injections can potentially induce robust transgene expression in local joints and provide long-term relief of inflammation in RA patients. This may reduce the amount of systemic therapies needed for treatment while improving levels of chronic joint pain and swelling, and eventually, the joint destruction and disability associated with RA. In this study, we only investigated the role of PD-L1 *via* AAV gene delivery in preventing RA development and blocking the progression from early phase of RA. Further studies will be focused on enhancing PD-L1 expression and secretion, optimizing AAV vectors for maximal efficiency, and investigating the long-term efficiency of PD-L1 in blocking the flare-ups or established joint inflammation.

## Data availability statement

The original contributions presented in the study are included in the article/**Supplementary Material**. Further inquiries can be directed to the corresponding author.

## Ethics statement

The animal study was reviewed and approved by Institutional Animal Care and Use Committee at the University of North Carolina at Chapel Hill (UNC IACUC ID: 21-233.0).

## Author contributions

CL, RL, EW and SW contributed to conception and design of the study. WL, FW and MM organized the database. WL performed the statistical analysis. WL, JS and SF did the animal operations. WL wrote the first draft of the manuscript. All authors contributed to the article and approved the submitted version.
