# Supplementary material for: Intra-articular delivery of AAV vectors encoding PD-L1 attenuates joint inflammation and tissue damage in a mouse model of rheumatoid arthritis
Source: Front Immunol. 2023 Mar 3;14:1116084. doi: 10.3389/fimmu.2023.1116084 (PMC10021025; doi:10.3389/fimmu.2023.1116084)
Supplement: Supplementary file 1 [file DataSheet_1.docx]

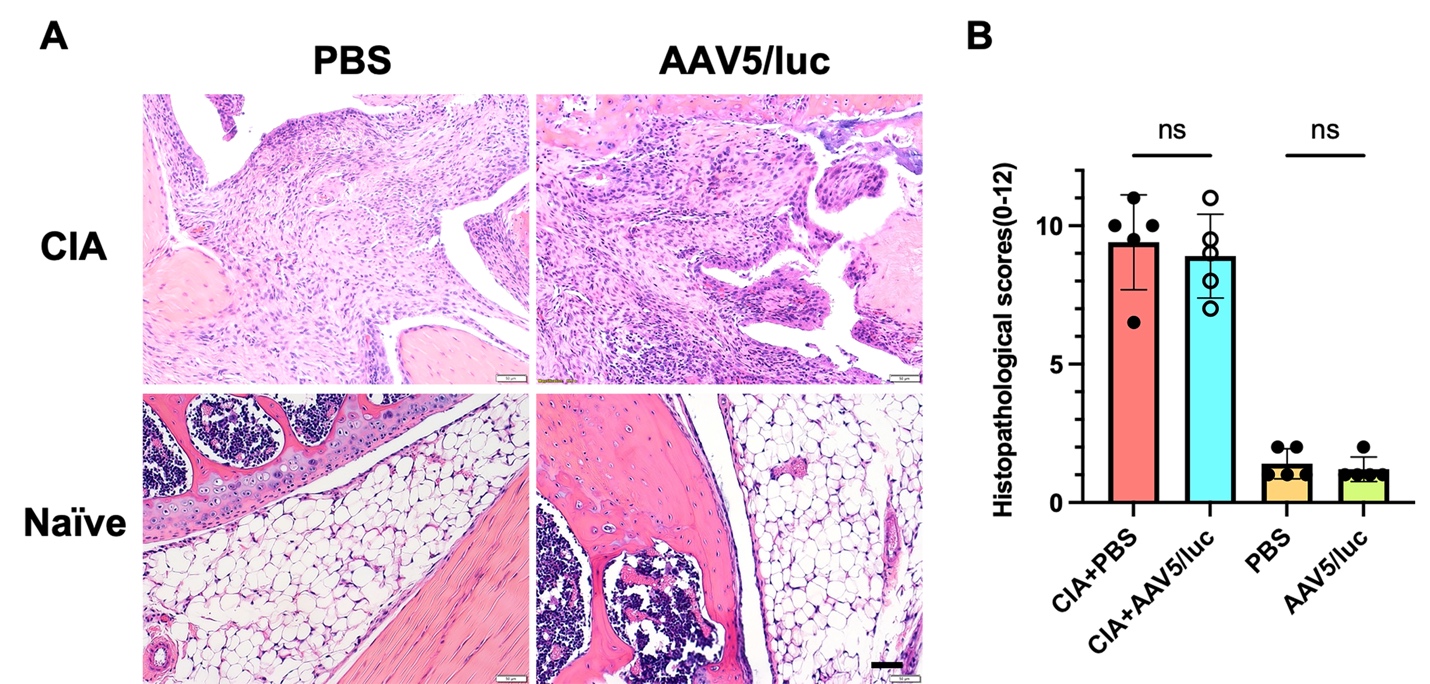


**Fig. S1** **Histology score of joints injected with PBS and AAV5/luc.** A, Representative images of H&E staining from the CIA mice and naïve mice knees injected with PBS or AAV5/luc at week 7 are shown (n=5, bar =50μm). B, Histopathological scores of CIA and naïve mice injected with PBS and AAV5/luc respectively(n=5). Mean values were shown with standard derivation. Data was analyzed using two-tailed unpaired student’s t test.


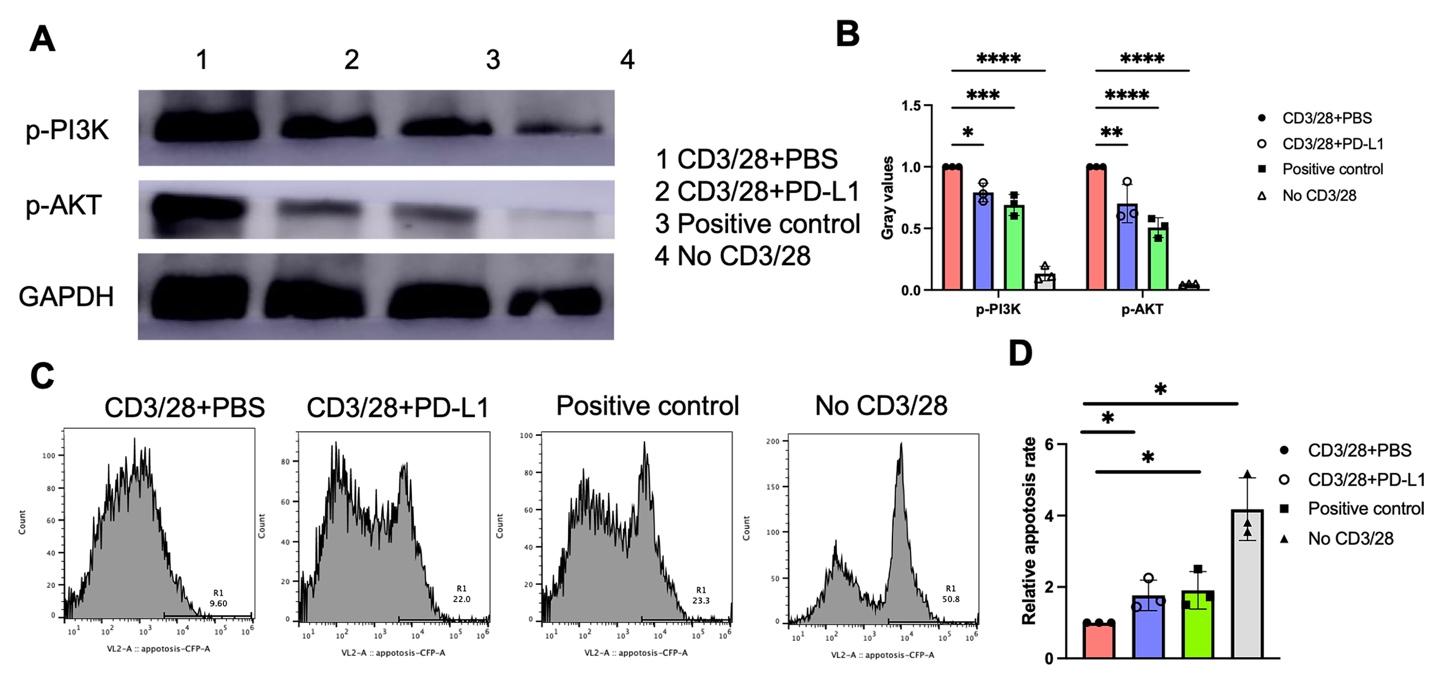


**Fig. S2** A, Western blot of phosphorylated PI3K and AKT protein expression. T cells were activated by CD3/28 and incubated with or without 5 μg/ml PD-L1 for 48h, the cell lysate was collected. B, Gray value of protein expression in each group relative to CD3/28+PBS group. Mean values are shown with standard derivation. Data was analyzed using one-way ANOVA followed by Bonferroni multiple comparison test for comparisons. *, p < 0.05, **, p < 0.01, ***, p < 0.005, ****, p < 0.001. C, T cell apoptosis. Purified splenic T cells were co-cultured with PBS, PD-L1 in the presence of anti-CD3/anti-CD28 for 72h. The apoptosis rate of positively stained cells was analyzed with flow cytometry. D, Summary data of T cell apoptosis. The result was normalized to T cell apoptosis rate in the group with CD3/28+PBS (n=3). Mean values are shown with standard derivation. Data was analyzed using one-way ANOVA followed by Bonferroni multiple comparison test for comparisons. *, p < 0.05.


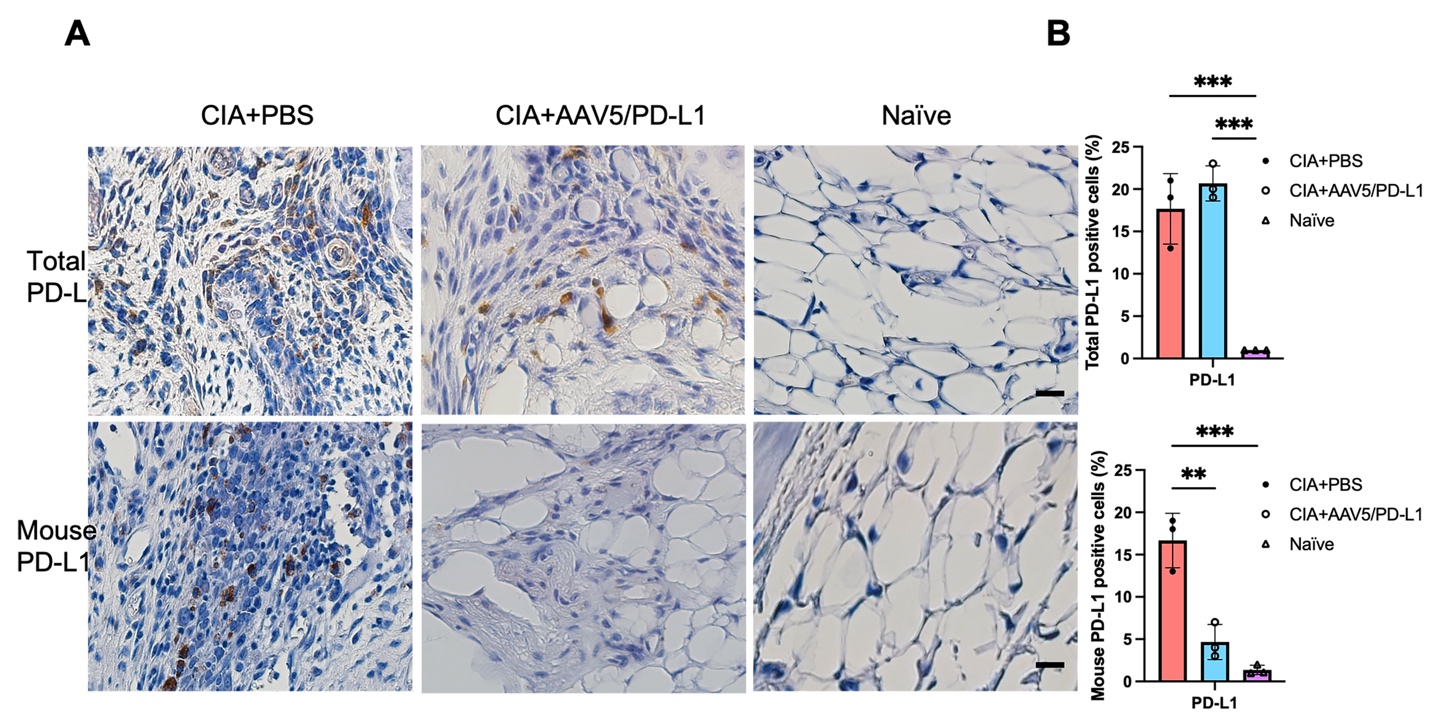


**Fig. S3 PD-L1 expression level in different groups.** A, Representative images of in situ immunohistochemical visualization of total PD-L1(both mouse PD-L1 and human PD-L1) expression and mouse PD-L1 from the CIA mouse knees injected with PBS or AAV5/PD-L1 and naïve mice at week 7(n=3, bar =20μm). B, Positive cell percentage of total PD-L1 and mouse PD-L1 (n=3). Mean values were shown with standard derivation. Data were analyzed using one-way ANOVA followed by Bonferroni multiple comparison test for group comparisons. **, p < 0.01, ***, p < 0.005, ****, p < 0.001.


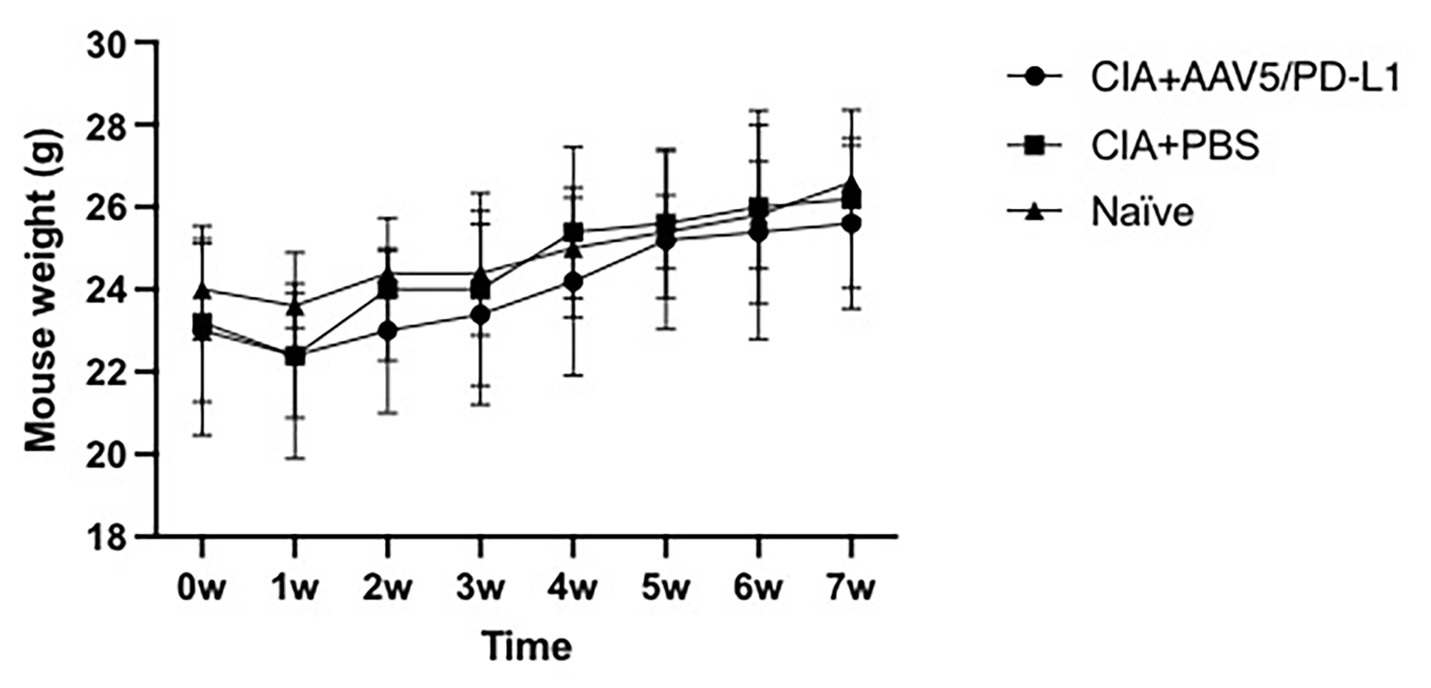


**Fig. S4 Mouse weight after the booster dose of immunization.** From 0w to 7w after the booster dose, the mice weight was monitored(n=10), no mice died or went through significant weight loss. Mean values were shown with standard derivation (n=10). Data at each time point was analyzed using one-way ANOVA followed by Bonferroni multiple comparison test for group comparisons.  Among the groups of CIA+PBS, CIA+AAV5/PD-L1 and naïve mice, no significant difference of weight in each time point was detected.

**
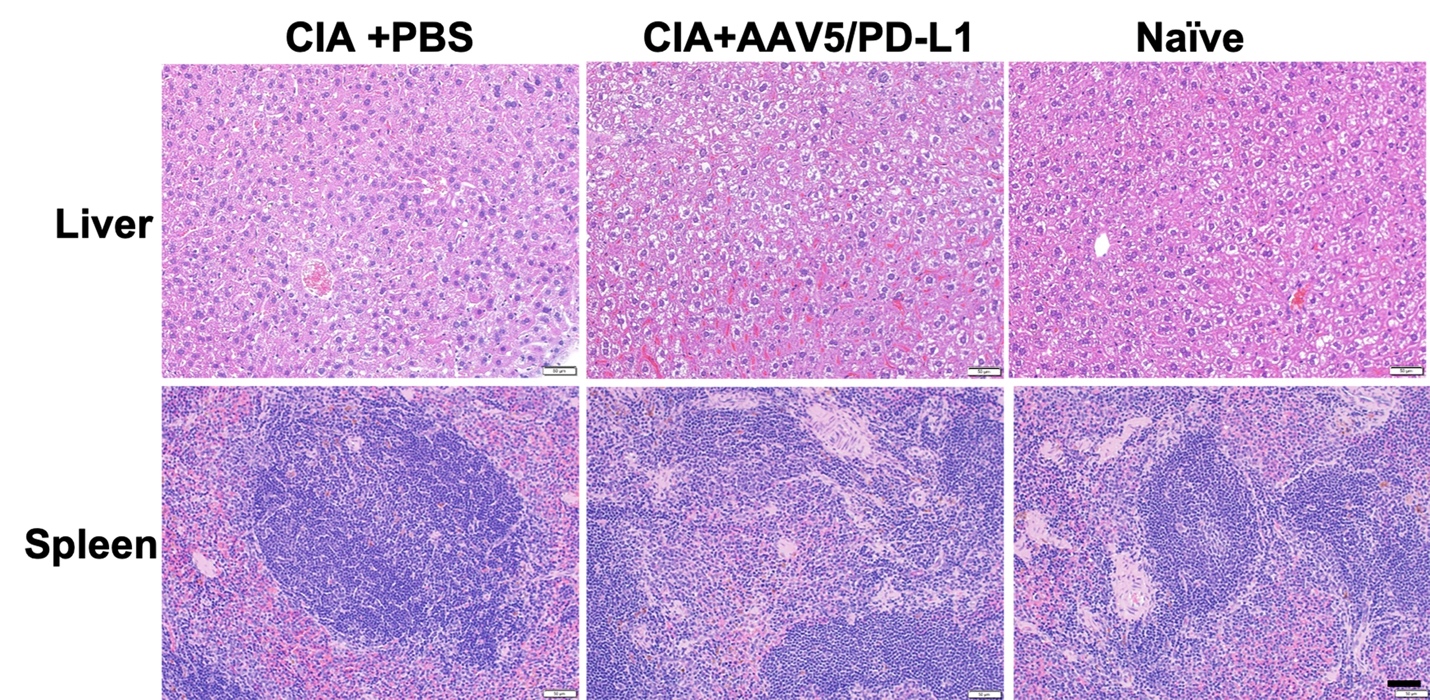
**

**Fig. S5 Hematoxylin-Eosin staining of liver and spleen.** The hepatocytes from treated group, untreated group, and wildtype mice (bar=50μm, n=3) were normal in shape and arrangement, the hepatocyte nuclei looked normal in a round shape, with one or two nucleoli. No macrovesicular steatosis, necrosis, or apoptotic hepatocytes were observed, indicating no signs of hepatitis, hepatocellular damage, or carcinoma.

**
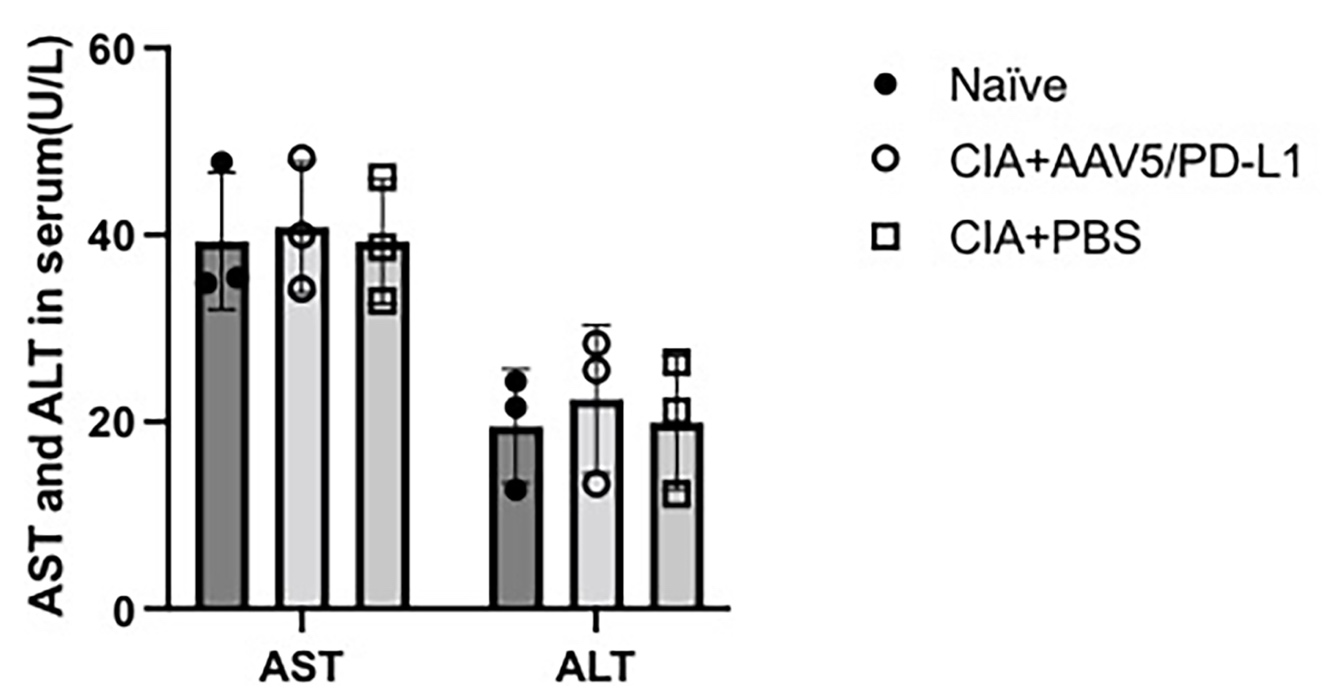
**

**Fig. S6 AST and ALT level in blood at 7w.** At 7w, the serum was collected, the average AST and ALT were calculated. Mean values were shown with standard derivation(n=3). Data was analyzed using one-way ANOVA followed by Bonferroni multiple comparison test for group comparisons. There was no significant difference between each group.


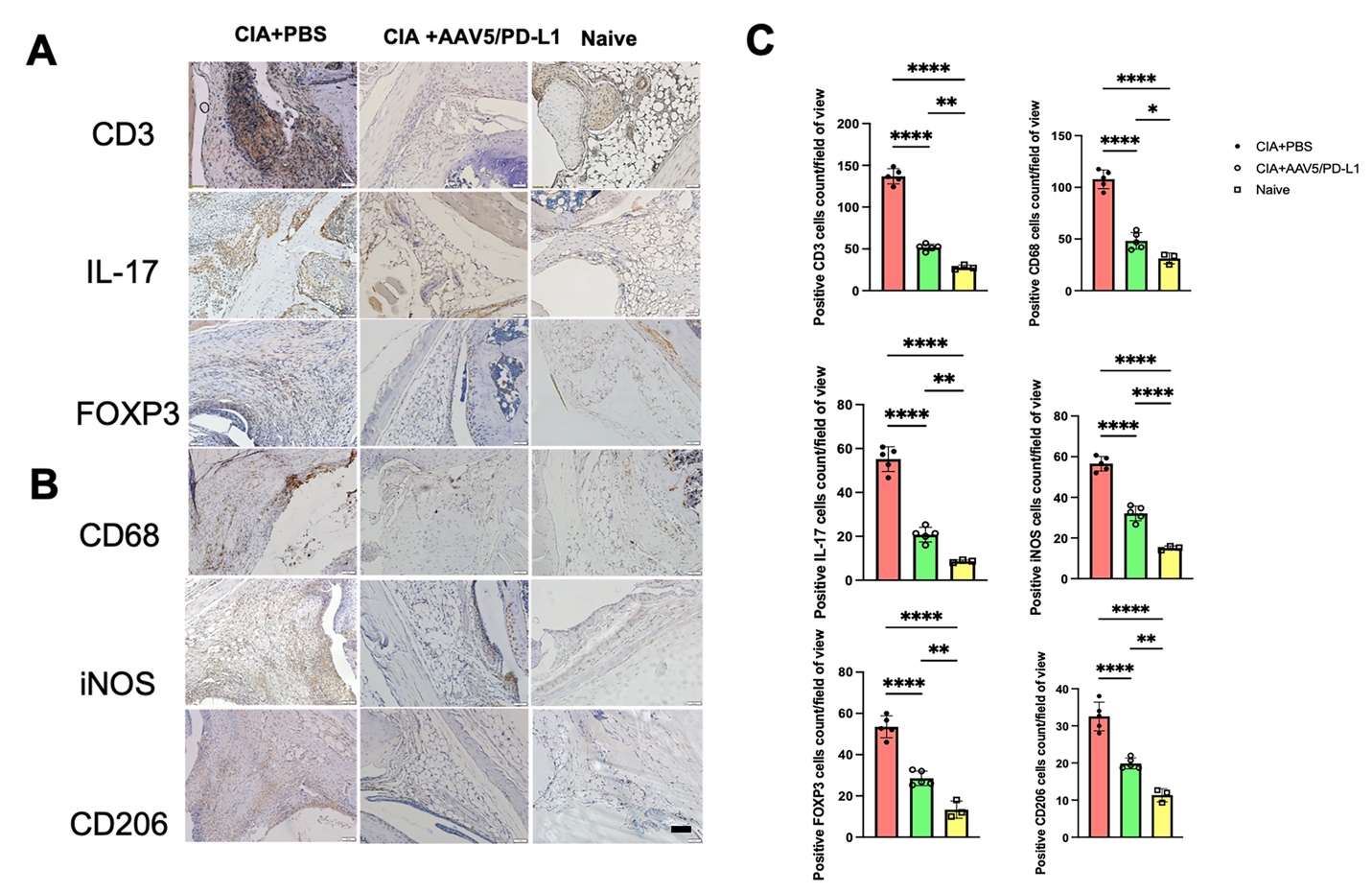


**Fig. S7 Infiltration of T-cell and macrophage in the synovium for therapeutic treatment at 7w.** A, Representative images of in situ immunohistochemical visualization of T cells by CD3, IL-17, FOXP3 staining (bar=50μm); B, Representative images of in situ immunohistochemical visualization of macrophages by CD68, iNOS, CD206 staining (bar=50μm). C, Positive cell counts per field of view of CD3, IL-17, FOXP3, CD68, iNOS and CD206(n=5). Mean values were shown with standard derivation. Data were analyzed using one-way ANOVA followed by Bonferroni multiple comparison test for group comparisons. *, p < 0.05, **, p < 0.01, ***, p < 0.005, ****, p < 0.001.
